# Effect of medial open wedge high tibial osteotomy on progression of patellofemoral osteoarthritis

**DOI:** 10.1186/s43019-022-00170-2

**Published:** 2022-10-23

**Authors:** Bo-Ram Na, Hong-Yeol Yang, Jae-Woong Seo, Chang-Hyun Lee, Jong-Keun Seon

**Affiliations:** 1 Department of Orthopedic Surgery, Chonnam National University Medical School and Hospital, 322 Seoyang-Ro, Hwasun-Gun, Chonnam, 58128 Republic of Korea; 2 Department of Orthopedic Surgery, Segyero Hospital, Gwangju, Korea; 3 Department of Orthopedic Surgery, Bitgaram General Hospital, Naju, Korea

**Keywords:** Knee, High tibial osteotomy, Patellofemoral joint, Cartilage lesion

## Abstract

**Purpose:**

This study aimed to investigate the effect of medial open wedge high tibial osteotomy (MOWHTO) on patellofemoral joint osteoarthritis (PF OA) progression and its outcome according to the degree of preexisting PF OA.

**Materials and methods:**

Patients who underwent biplane MOWHTO between January 2006 and December 2018 were retrospectively reviewed. The patients were divided into two groups according to the degree of PF OA: non-PF OA [Kellgren–Lawrence (K–L) grade 0–1] and PF OA (K–L grade 2–3). Propensity score matching was performed between the two groups, and comparative analysis was performed on clinical scores and radiographic parameters and grade.

**Results:**

After propensity score matching, 83 patients were selected for each group. At postoperative follow-up, clinical scores were improved significantly compared with preoperative scores in both groups; however, there were no significant differences between the groups. There were also no significant differences between the two groups in radiographic parameters. The radiographic grade of PF OA indicated a slight progression in osteoarthritis in both groups; however, PF OA tended to progress further in the PF OA group.

**Conclusions:**

MOWHTO did not result in significant differences in outcomes at postoperative follow-up; however, preexisting PF OA contributed to PF OA progression after MOWHTO.

## Introduction

Medial open wedge high tibial osteotomy (MOWHTO) is an effective surgical procedure for the treatment of medial compartment osteoarthritis of the knee and the correction of lower extremity malalignment [1, 2]. Owing to favorable clinical results and improved surgical techniques, MOWHTO is becoming increasingly popular [3–5].

Although MOWHTO has shown favorable clinical results, there are also reports that MOWHTO may adversely affect the patellofemoral joint. Several studies have reported that MOWHTO leads to patella height and patellofemoral alignment changes, consequently resulting in increased patellofemoral contact pressure [6–9]. In particular, overcorrection of the varus alignment has also been reported to affect the progression of patellofemoral joint osteoarthritis (PF OA) [10–12]. However, it is difficult to definitively conclude that MOWHTO contributes to the deterioration of the articular cartilage in the patellofemoral joint. Various factors could affect the progression of PF OA [13]. Recently, preexisting cartilage lesions have been identified as a factor affecting PF OA after MOWHTO. In general, preexisting cartilage lesions in joints are known to act as a leading factor in the development of arthritis by concentrating stress at the rim of the defect [14]. Several studies examining the effect of MOWHTO on the patellofemoral joint through arthroscopic evaluation reported that the articular cartilage in the patellofemoral joint deteriorated over time due to MOWHTO [7, 11, 12, 15]. However, these studies were not comparative studies based on the presence of preexisting lesions in the articular cartilage. To the best of our knowledge, limited studies have examined the progression of PF OA after MOWHTO according to the degree of preexisting osteoarthritis in the patellofemoral joint.

The purpose of this study was to investigate the effect of MOWHTO on the progression of PF OA and its clinical outcome according to the degree of preexisting PF OA. We hypothesized that MOWHTO would contribute to the progression of PF OA when the preexisting PF OA is more severe and that the clinical outcomes of MOWHTO in patients with more severe preexisting PF OA would be inferior.

## Materials and methods

**Fig. 1:**
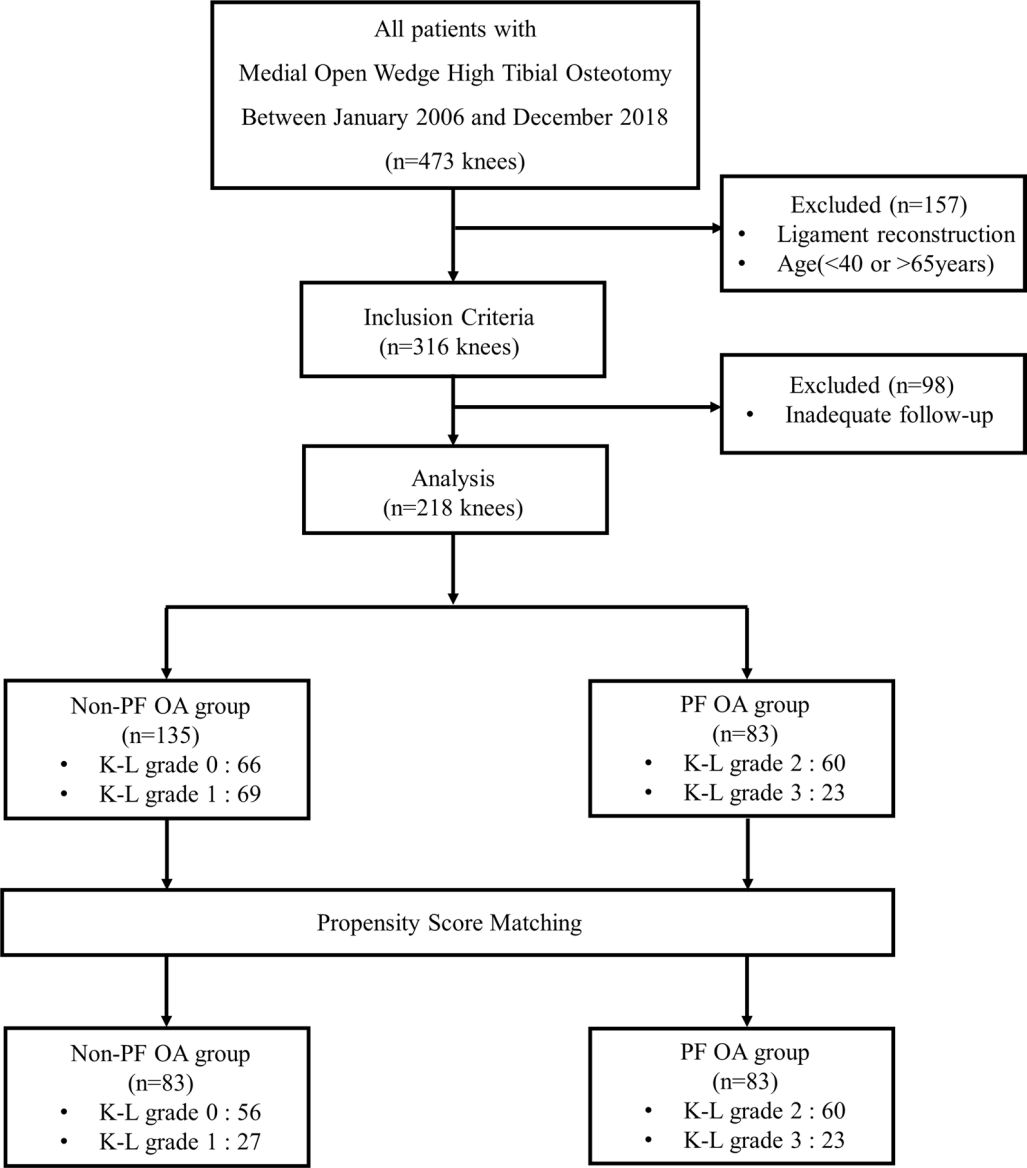
Flowchart of patient inclusion in the study. PF OA: patellofemoral osteoarthritis, *K–L* Kellgren–Lawrence

The present investigation was approved by the institutional review board before it commenced. Due to the retrospective nature of the study and the use of anonymized patient data, requirements for informed consent were waived. Data from 218 consecutive patients, who underwent biplane MOWHTO performed by a single orthopedic surgeon in a single institution between January 2006 and December 2018, were retrospectively reviewed. Surgical indications for MOWHTO were as follows: age > 40 and < 65 years; medial compartment osteoarthritis and varus malalignment; activity-related medial-sided knee pain; good range of motion (arc of motion > 100° and flexion contracture < 15°); and no joint instability. MOWHTO was not indicated for patients with complaints of anterior knee pain associated with activities such as squatting and stair climbing or descending. Individuals with a history of surgical treatment of the knee, surgical site infection, additional surgical procedures on the same knee during the follow-up period, and the same subsequent surgical procedure on the opposite knee during the follow-up period were excluded from the study. Patients were divided into two groups according to the degree of PF OA. The distribution of patients in the two groups was as follows: non-PF OA group, 135 patients with Kellgren–Lawrence (K-L) grade 0 or 1; PF OA group, 83 patients with K–L grade 2 or 3. To control for potential confounding variables, the patients from both groups were matched through propensity score matching. Finally, a total of 83 patients were included for propensity score-matched analysis in each group (Fig. 1).

### Surgical procedure

For all patients, preoperative surgical planning to achieve a proper correction angle was performed according to the methods described by Miniaci and Dugdale, which involve the realignment of the mechanical axis to the Fujisawa point [16–18]. Before the osteotomy procedure, diagnostic arthroscopy was performed, and the status of the articular cartilage was thoroughly evaluated. No cartilage-specific procedures such as debridement and chondroplasty were performed on lesions of the patellofemoral joint cartilage. After arthroscopic assessment, biplane MOWHTO was performed following a technique developed by the Arbeitsgemeinschaft für Osteosynthesefragen (AO) knee expert group [5]. To expose the medial proximal tibia, an oblique skin incision, approximately 6–7 cm in length, was made from 1 cm below the joint line to the pes anserinus tendons between the tibial tuberosity and the posteromedial border of the tibia. The long fibers of the medial collateral ligament were released as much as necessary to expose the posteromedial crest of the tibia. Two starting guide wires for transverse osteotomy were inserted parallel from the upper border of the pes anserinus tendons toward the upper portion of the fibular head. Before transverse osteotomy, a separate oblique vertical osteotomy was performed in the coronal plane 1 cm behind the tibial tuberosity. In the anterior third, an ascending cut was performed behind the tibial tuberosity, creating a 130° angle to the posterior plane of osteotomy. Transverse osteotomy was subsequently initiated using an oscillating saw along the two guide wires leaving the lateral most at 1 cm of the proximal tibia as a hinge. The osteotomy site was opened gradually using several chisels and a spreader device. After the desired correction was achieved, a locking plate was applied and fixed to the medial proximal tibia over the osteotomy site.

Postoperatively, the patients were instructed to begin crutch-assisted progressive weight-bearing ambulation and hinged knee brace-assisted knee range of motion exercise as tolerated. Six weeks after surgery, all patients were encouraged to discontinue the use of crutches and remove the knee brace.

### Evaluation

**Fig. 2:**
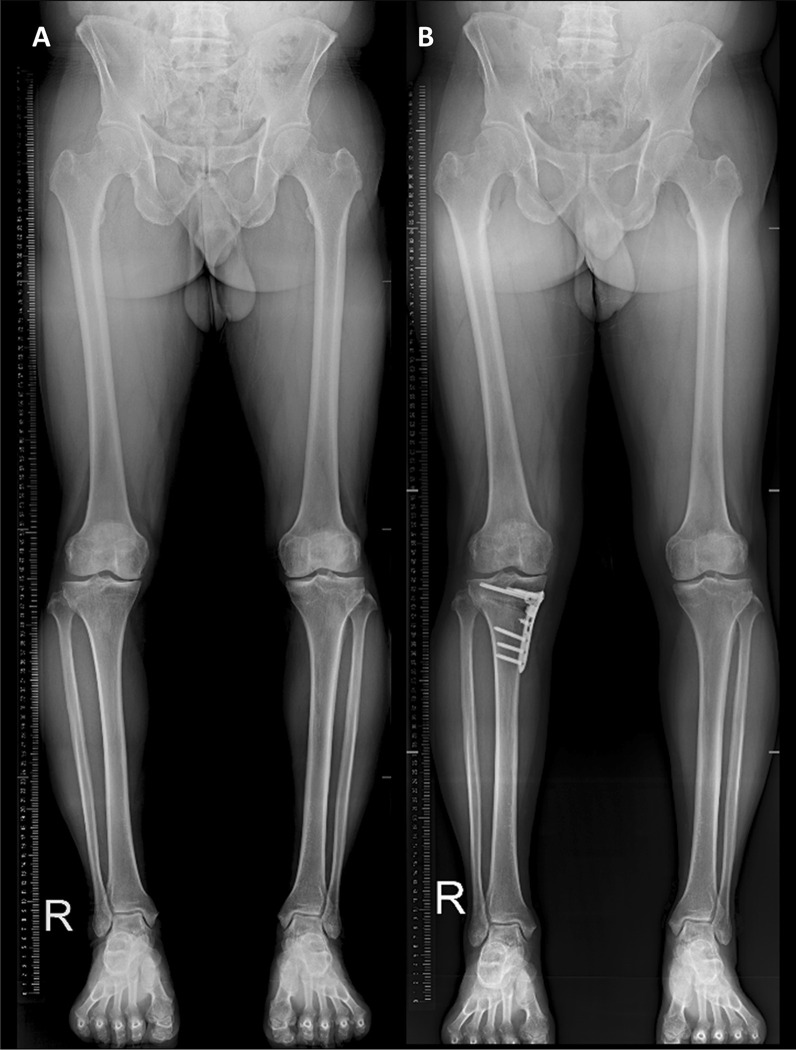
Anterior–posterior standing hip–ankle radiographs of a 55-year-old man. **A** Preoperative radiograph showing varus limb alignment. **B** Postoperative radiograph showing correction of varus malalignment and valgus limb alignment after medial open wedge high tibial osteotomy

Comparative analysis of clinical outcomes and radiographic parameters and grade was performed. Clinical outcomes were assessed preoperatively and postoperatively using various patient-reported knee rating scales, including a visual analog scale (VAS), the Western Ontario and McMaster Universities Osteoarthritis Index (WOMAC), and Knee Society score (KSS) [19]. Various radiographic parameters possibly associated with the preexisting condition of the patellofemoral joint, including mechanical axis, weight-bearing line ratio, and posterior tibial slope, were evaluated [20]. The radiographic assessment was based on the anterior–posterior, lateral, and Merchant views of the knee and anterior–posterior standing hip-ankle radiographs (Fig. 2). The grade of osteoarthritis was radiographically assessed using the K–L grading system [21]. Clinical outcomes and radiographic parameters and grade were assessed annually at postoperative follow-up (average follow-up period of 5.63 years). Two orthopedic surgeons who were not involved in the surgery measured all radiographic parameters at an interval of 6 weeks and were blinded to one another’s measurements. Arthroscopic assessment was performed at the time of the initial operation. All arthroscopic measurements and plane radiographs were obtained immediately after the surgery by the orthopedic surgeon who performed MOWHTO.

### Statistical analysis

Propensity scores were calculated by logistic regression analysis of the covariates (gender, age, follow-up duration, preoperative range of motion, BMI, preoperative mechanical axis, and preoperative posterior tibial slope). The patients were matched using the nearest neighbor technique, with a predefined caliper width equal to 0.2 of the standard deviation of the logit of the propensity score. The assumption of normality of the numerical data was verified by the Shapiro–Wilk test. The independent sample *t*-test or Mann–Whitney *U* test was used to compare the two groups in terms of numerical demographic characteristics, preoperative and postoperative measurements, and surgical data depending on whether the data were normally distributed. The paired *t*-test was used to analyze intragroup differences before and after surgery. For categorical data, the chi-squared test was used to assess differences in the progression of osteoarthritis after surgery between the two groups. Statistical analysis was performed using SPSS version 22.0 (IBM Corp, Armonk, NY, USA) for Windows (Microsoft Corporation, Redmond, WA, USA). Differences with *p* < 0.05 were considered to be statistically significant.

## Results

This study included 83 patients in the non-PF OA group and 83 patients in the PF OA group. Baseline characteristics, including preoperative average clinical scores, deformities, and age at operation in the PF OA group, were not significantly different from those in the non-PF OA group (Table 1). Based on radiological results, postoperative radiographic parameters were not significantly different between the two groups (Table 2). At postoperative follow-up, patients in both the non-PF OA and PF OA groups exhibited a slight progression in osteoarthritis based on the K–L classification. PF OA tended to progress further in the PF OA group (61/83 patients, 73.49%) than in the non-PF OA group (43/83 patients, 51.81%) (Table 3). Clinical outcomes (VAS, WOMAC, KSS pain, and KSS function) demonstrated significant overall improvement from baseline to the last follow-up, with no statistically significant differences between the two groups at each time point (Table 4).

**Table 1:** Comparison of baseline characteristics

Variable	Non-PF OA group (*n* = 83)	PF OA group (*n* = 83)	*p*-Value
Gender (M/F)	23/60	22/61	0.500
Age (years)	54.83 ± 6.77	55.79 ± 5.81	0.223
F/U duration (years)	5.52 ± 3.07	5.73 ± 3.14	0.653
Range of motion (°)	134.2 ± 8.6	133.3 ± 9.6	0.602
BMI (kg/m^2^)	25.7 ± 3.1	25.1 ± 3.2	0.547
Mechanical axis (°)	5.89 ± 2.85	6.05 ± 2.66	0.550
Weight-bearing line ratio	0.23 ± 0.11	0.21 ± 0.12	0.379
Posterior tibial slope (°)	9.33 ± 3.79	10.23 ± 3.39	0.307
Preoperative VAS	4.57 ± 1.82	4.67 ± 2.35	0.141
Preoperative WOMAC	41.41 ± 17.27	43.09 ± 23.69	0.324
Preoperative KSS pain	28.22 ± 11.66	27.22 ± 11.08	0.228
Preoperative KSS function	64.64 ± 17.25	65.68 ± 15.48	0.875
Kellgren–Lawrence grade			0.000
Grade 0	56 (67.5%)	0 (0.0%)	
Grade 1	27 (32.5%)	0 (0.0%)	
Grade 2	0 (0.0%)	60 (72.3%)	
Grade 3	0 (0.0%)	23 (27.7%)	
Preoperative ICRS grade			0.000
Grade 0	60 (72.3%)	0 (0.0%)	
Grade 1	23 (27.7%)	2 (2.4%)	
Grade 2	0 (0.0%)	53 (63.9%)	
Grade 3	0 (0.0%)	28 (33.7%)	

The *p*-values are of intergroup comparisons, with *p* < 0.05 indicating significance. *PF OA* patellofemoral osteoarthritis, *ICRS* International Cartilage Repair Society, *VAS* visual analog scale, *WOMAC* Western Ontario and McMaster Universities Osteoarthritis Index, *KSS* Knee Society score

**Table 2:** Comparison of radiological outcomes

Parameters	Non-PF OA group (*n* = 83)	PF OA group (*n* = 83)	*p*-Value
Mean mechanical axis (°)			
Preoperative	5.89 ± 2.85	6.05 ± 2.66	0.550
Last follow-up	−0.96 ± 2.08	−0.56 ± 3.15	0.393
Correction angle	6.85 ± 3.11	6.62 ± 3.65	0.376
Mean weight-bearing line ratio			
Preoperative	0.23 ± 0.11	0.21 ± 0.12	0.379
Last follow-up	0.53 ± 0.10	0.53 ± 0.13	0.849
Mean posterior tibial slope (°)			
Preoperative	9.83 ± 3.79	10.23 ± 3.39	0.307
Last follow-up	10.89 ± 4.16	11.12 ± 3.40	0.742
ΔPTSA	1.06 ± 3.46	0.88 ± 4.04	0.696

The *p*-values are of intergroup comparisons, with *p* < 0.05 indicating significance. *PF OA* patellofemoral osteoarthritis, *correction angle* difference in mechanical axis angle, *ΔPTSA* difference in posterior tibial slope angle

**Table 3:** Change of K–L grade at follow-up in patellofemoral joint

Grade change	Non-PF OA group (*n* = 83)	PF OA group (*n* = 83)	*p*-Value
No change	40 (48.19%)	22 (26.50%)	0.003
OA aggravation	43 (51.81%)	61 (73.49%)	0.003
Grade change 1	31 (37.35%)	47 (56.63%)	0.010
Grade change ≥ 2	12 (14.46%)	14 (16.87%)	0.416

The *p*-values are of intergroup comparisons, with *p* < 0.05 indicating significance. *K–L* Kellgren–Lawrence, *PF OA* patellofemoral osteoarthritis, *OA* osteoarthritis

**Table 4:** Comparison of clinical outcomes

Parameters	Non-PF OA group (*n* = 83)	PF OA group (*n* = 83)	*P*-value
VAS			
Preoperative	4.57 ± 1.82	4.67 ± 2.35	0.141
Postoperative	1.89 ± 1.16	1.86 ± 1.50	0.454
WOMAC			
Preoperative	41.41 ± 17.27	43.09 ± 23.69	0.324
Postoperative	17.01 ± 12.00	16.20 ± 14.31	0.438
KSS pain			
Preoperative	28.22 ± 11.66	27.22 ± 11.08	0.228
Postoperative	43.25 ± 9.24	44.23 ± 11.18	0.471
KSS function			
Preoperative	64.64 ± 17.25	65.68 ± 15.48	0.875
Postoperative	85.18 ± 15.57	84.62 ± 16.67	0.563

The *p*-values are of intergroup comparisons, with *p* < 0.05 indicating significance. *VAS* visual analog scale, *WOMAC* Western Ontario and McMaster Universities Osteoarthritis Index, *KSS* Knee Society score

It is clinically important to estimate whether the clinical outcome score improved beyond a minimally clinically important difference (MCID), specifically the change reported by patients who identified themselves as minimally better or minimally worse as a result of the surgical procedure [22]. According to previous studies, MCIDs for the VAS, WOMAC, and KSS pain and function scores were 1.99, 22.6, 3.0, and 5.6 points, respectively [22, 23]. In the current study, the total VAS, WOMAC, and KSS pain and function scores were improved by 2.67, 24.40, 13.04, and 20.54 points, respectively, in the non-PF OA group, and 2.81, 26.89, 17.01, and 18.94 points, respectively, in the PF-OA group (Table 5). In both groups, the mean improvement in the total VAS, WOMAC, and KSS pain and function scores was more than the MCID. No complications, such as deep infection, loosening, or osteolysis requiring revision surgery, occurred in either group.

**Table 5:** MCID values for the PROMs

PROMs	Non-PF OA group average change (%, above MCID)	PF OA group average change (%, above MCID)	MCID
VAS	2.67 (79.5%)	2.81 (83.1%)	1.99
WOMAC	24.40 (66.3%)	26.89 (70.3%)	22.6
KSS pain	13.04 (92.8%)	17.01 (100%)	3.0
KSS function	20.54 (95.2%)	18.94 (100%)	5.6

*PROMs* patient-reported outcome measures, *MCID* minimal clinically important difference using the ROC curve anchor method, *VAS* visual analog scale, *WOMAC* Western Ontario and McMaster Universities Osteoarthritis Index, *KSS* Knee Society score

## Discussion

**Fig. 3:**
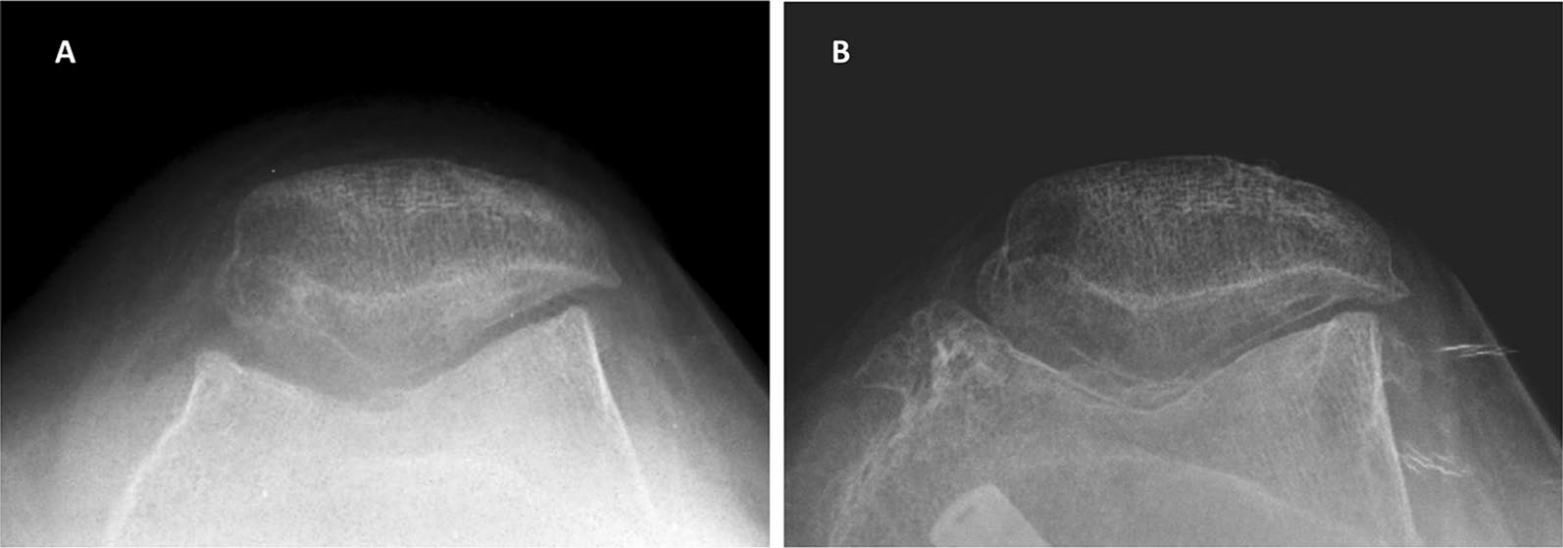
Merchant view radiographs of a 58-year-old woman (case 1). **A** Preoperative radiograph showing Kellgren–Lawrence grade 2 patellofemoral joint osteoarthritis. **B** Follow-up radiograph 5 years after surgery showing Kellgren–Lawrence grade 4 patellofemoral joint osteoarthritis

**Fig. 4:**
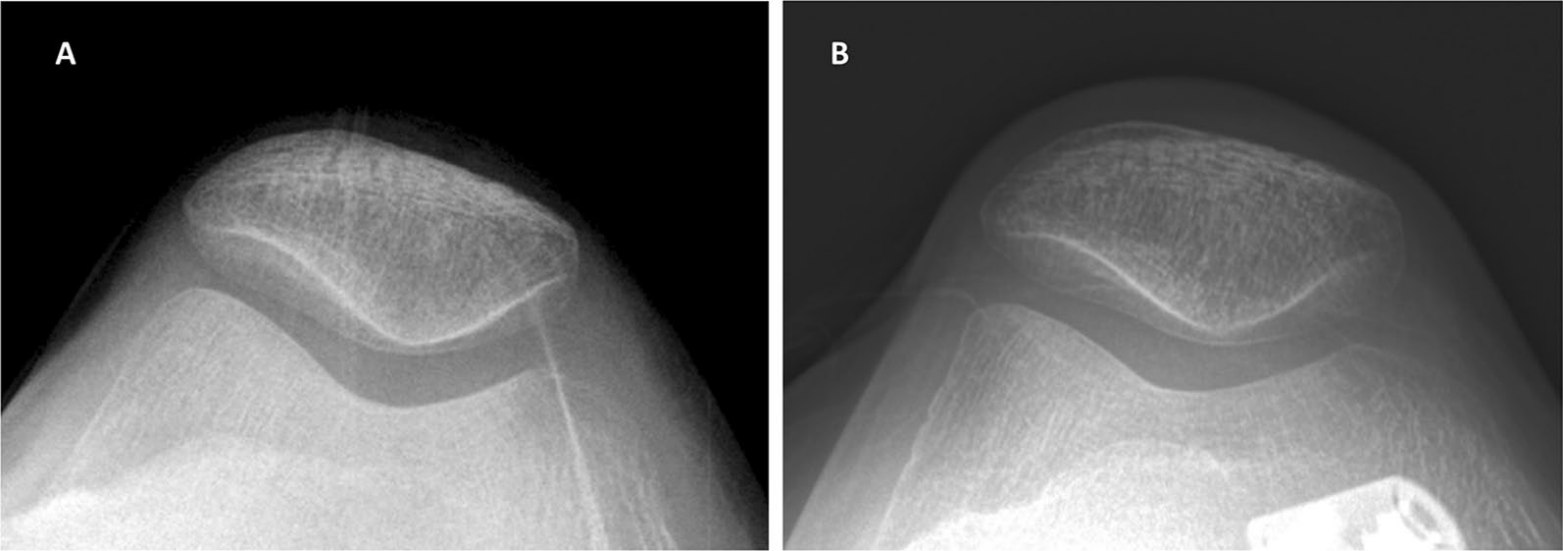
Merchant view radiographs of a 56-year-old woman (case 2). **A** Preoperative radiograph showing no patellofemoral joint osteoarthritis. **B** Follow-up radiograph 5 years after surgery showing no patellofemoral joint osteoarthritis

Radiological evaluation revealed that PF OA tended to progress further in the PF OA group than in the non-PF OA group (Figs. 3 and 4). The differences in clinical outcomes and radiographic parameters between the PF OA and non-PF OA groups were not statistically significant in this study. Our study results support our hypothesis that MOWHTO would contribute to the progression of PF OA when the preexisting PF OA is more severe, but did not support our hypothesis that patients with more severe preexisting PF OA would have worse clinical outcomes after MOWHTO.

The main finding of the current study was that MOWHTO contributed to the progression of PF OA in patients with more severe preexisting PF OA; however, it was not directly associated with clinical outcomes. Therefore, caution is needed when performing MOWHTO in the presence of preexisting PF OA. MOWHTO is known to negatively affect the patellofemoral joint owing to changes in the patellar position. A decreased patellar height and an altered patellofemoral alignment could increase the patellofemoral contact pressure, consequently increasing the risk of progression of osteoarthritis [6, 8, 9, 24]. Several studies have performed an arthroscopic assessment of the progression of PF OA resulting from MOWHTO [7, 11, 12, 15]. However, it is difficult to conclude that MOWHTO is the most important contributor to the progression of PF OA.

In theory, although the increased contact pressure of the patellofemoral joint may lead to the progression of osteoarthritis in the affected joint, many variables need to be considered. Recent studies have investigated relevant factors that can influence the progression of PF OA after MOWHTO. The progression of cartilage degeneration may be attributed to normal age-dependent joint degeneration, as noted in previous studies [7, 11, 12]. Yoon et al. reported that overcorrection (i.e., a postoperative weight-bearing line ratio > 66.3%) could lead to the further progression of patellofemoral joint degeneration after MOWHTO [12]. Similarly, Tanaka et al. reported that lesions in the cartilage of the patellofemoral joint tended to progress after MOWHTO in patients with a medial opening gap ≥ 13 mm or a change in the medial proximal tibial angle ≥ 9° [11]. Focal articular cartilage defects are also known to be a predisposing factor for osteoarthritis [14]. Preexisting cartilage lesions in the patellofemoral joint can be a risk factor that contributes to the further progression of PF OA. In this study, PF OA tended to progress further in the presence of preexisting PF OA compared with a normal patellofemoral joint. Owing to methodological differences, there may be limitations in applying, interpreting, and comparing the results from previous studies and the present study. Nevertheless, other factors of PF OA progression, excluding preexisting PF OA, did not differ between the two groups in this study. Although the present study did not examine all factors that can influence the progression of patellofemoral joint degeneration after MOWHTO, preexisting PF OA, which may be a risk factor for PF OA progression, would be an important consideration in performing MOWHTO.

In the present study, subgroup analysis showed that clinical outcomes were not significantly correlated with the degree of preexisting PF OA, which may be attributed to inaccuracies in assessing the status of the patellofemoral joint. The clinical symptoms of PF OA can vary, including anterior knee pain and patellofemoral dysfunction. Usually, physicians evaluate the status of the patellofemoral joint by physical examination (comprising a patellar compression test and an assessment for the Clarke sign) or radiographic examination [25]. However, because these tools are difficult to standardize and rely on physician experience and the interpretation of patient responses, their utility in measuring outcomes is limited. For this reason, various scoring systems have been developed for assessing the knee joint; however, not many of them are suitable for assessing the condition of the patellofemoral joint. In addition, limited studies have reported the use of patellofemoral scoring tools that can specifically assess anterior knee pain or patellofemoral dysfunction; thus, a properly validated and reliable patellofemoral-specific scoring system has not been established [26, 27]. For this reason, the VAS, WOMAC, and KSS pain and function scales used in our study may not accurately reflect the clinical outcomes of patients with PF OA. Although this study did not show that the progression of PF OA was correlated with specific clinical features, it should be noted that PF OA progression is possible when MOWHTO is performed.

Some limitations of the present study should be considered. First, the progression of PF OA was graded according to radiographic findings. The grade of osteoarthritis was first assessed by radiography, which was validated by arthroscopic assessment while performing the operation. However, postoperative assessment was performed only radiologically. Even radiographic evaluations may not provide accurate information about the status of the patellofemoral joint [28]. Accordingly, postoperative arthroscopic assessment in further studies is required. Second, a clinical scoring system to evaluate patellofemoral joint arthritis has not been established, and the VAS, WOMAC, and KSS pain and function scales used in our study are insufficient. Research to identify scoring systems that better reflect the patellofemoral status, including the Feller, Kujala, and Samsung Medical Center (SMC) patellofemoral scoring systems, is warranted [26, 27, 29]. Third, the mean value of the posterior tibial slope was rather high and was increased after surgery. Although there were no statistically significant differences between the two groups, a pathologic posterior tibial slope can lead to extension deficit and affect clinical outcomes [30].

## Conclusions

MOWHTO did not result in significant differences in clinical outcomes and radiographic parameters at follow-up; however, preexisting PF OA contributed to the progression of PF OA after MOWHTO. Taken together, the results of this study suggest that caution is needed when performing MOWHTO in the presence of preexisting PF OA.

## Data Availability

The datasets during and/or analysed during the current study available from the corresponding author on reasonable request.

## References

[1] Floerkemeier S, Staubli AE, Schroeter S, Goldhahn S, Lobenhoffer P (2013). Outcome after high tibial open-wedge osteotomy: a retrospective evaluation of 533 patients. Knee Surg Sports Traumatol Arthrosc.

[2] Sprenger TR, Doerzbacher JF (2003). Tibial osteotomy for the treatment of varus gonarthrosis. Survival and failure analysis to twenty-two years. J Bone Jt Surg Am..

[3] Blackman AJ, Krych AJ, Engasser WM, Levy BA, Stuart MJ (2015). Does proximal tibial osteotomy with a novel osteotomy system obtain coronal plane correction without affecting tibial slope and patellar height?. Knee Surg Sports Traumatol Arthrosc.

[4] Cotic M, Vogt S, Feucht MJ, Saier T, Minzlaff P, Hinterwimmer S, Imhoff AB (2015). Prospective evaluation of a new plate fixator for valgus-producing medial open-wedge high tibial osteotomy. Knee Surg Sports Traumatol Arthrosc.

[5] Lobenhoffer P, Agneskirchner JD (2003). Improvements in surgical technique of valgus high tibial osteotomy. Knee Surg Sports Traumatol Arthrosc.

[6] Bin SI, Kim HJ, Ahn HS, Rim DS, Lee DH (2016). Changes in patellar height after opening wedge and closing wedge high tibial osteotomy: a meta-analysis. Arthroscopy.

[7] Goshima K, Sawaguchi T, Shigemoto K, Iwai S, Nakanishi A, Ueoka K (2017). Patellofemoral osteoarthritis progression and alignment changes after open-wedge high tibial osteotomy do not affect clinical outcomes at mid-term follow-up. Arthroscopy.

[8] Javidan P, Adamson GJ, Miller JR, Durand P, Dawson PA, Pink MM, Lee TQ (2013). The effect of medial opening wedge proximal tibial osteotomy on patellofemoral contact. Am J Sports Med.

[9] Yang JH, Lee SH, Nathawat KS, Jeon SH, Oh KJ (2013). The effect of biplane medial opening wedge high tibial osteotomy on patellofemoral joint indices. Knee.

[10] Cahue S, Dunlop D, Hayes K, Song J, Torres L, Sharma L (2004). Varus-valgus alignment in the progression of patellofemoral osteoarthritis. Arthritis Rheum.

[11] Tanaka T, Matsushita T, Miyaji N, Ibaraki K, Nishida K, Oka S, Araki D, Kanzaki N, Hoshino Y, Matsumoto T, Kuroda R (2019). Deterioration of patellofemoral cartilage status after medial open-wedge high tibial osteotomy. Knee Surg Sports Traumatol Arthrosc.

[12] Yoon TH, Choi CH, Kim SJ, Kim SH, Kim NH, Jung M (2019). Effect of medial open-wedge high tibial osteotomy on the patellofemoral joint according to postoperative realignment. Am J Sports Med.

[13] LaPrade RF (2017). Editorial commentary: is it the osteotomy that is causing the development of patellofemoral osteoarthritis or is it the normal progression of preexisting osteoarthritis?. Arthroscopy.

[14] Guettler JH, Demetropoulos CK, Yang KH, Jurist KA (2004). Osteochondral defects in the human knee: influence of defect size on cartilage rim stress and load redistribution to surrounding cartilage. Am J Sports Med.

[15] Kim KI, Kim DK, Song SJ, Lee SH, Bae DK (2017). Medial open-wedge high tibial osteotomy may adversely affect the patellofemoral joint. Arthroscopy.

[16] Miniaci A, Ballmer FT, Ballmer PM, Jakob RP (1989). Proximal tibial osteotomy. A new fixation device. Clin Orthop Relat Res..

[17] Dugdale TW, Noyes FR, Styer D (1992). Preoperative planning for high tibial osteotomy. The effect of lateral tibiofemoral separation and tibiofemoral length. Clin Orthop Relat Res..

[18] Fujisawa Y, Masuhara K, Shiomi S (1979). The effect of high tibial osteotomy on osteoarthritis of the knee. An arthroscopic study of 54 knee joints. Orthop Clin North Am..

[19] Flandry F, Hunt JP, Terry GC, Hughston JC (1991). Analysis of subjective knee complaints using visual analog scales. Am J Sports Med.

[20] Hohmann E, Bryant A, Reaburn P, Tetsworth K (2011). Is there a correlation between posterior tibial slope and non-contact anterior cruciate ligament injuries?. Knee Surg Sports Traumatol Arthrosc..

[21] Kellgren JH, Lawrence JS (1957). Radiological assessment of osteo-arthrosis. Ann Rheum Dis.

[22] Escobar A, Quintana JM, Bilbao A, Aróstegui I, Lafuente I, Vidaurreta I (2007). Responsiveness and clinically important differences for the WOMAC and SF-36 after total knee replacement. Osteoarthritis Cartilage.

[23] Tubach F, Ravaud P, Baron G, Falissard B, Logeart I, Bellamy N, Bombardier C, Felson D, Hochberg M, van der Heijde D, Dougados M (2005). Evaluation of clinically relevant changes in patient reported outcomes in knee and hip osteoarthritis: the minimal clinically important improvement. Ann Rheum Dis.

[24] Song IH, Song EK, Seo HY, Lee KB, Yim JH, Seon JK (2012). Patellofemoral alignment and anterior knee pain after closing- and opening-wedge valgus high tibial osteotomy. Arthroscopy.

[25] Malek MM, Mangine RE (1981). Patellofemoral pain syndromes: a comprehensive and conservative approach. J Orthop Sports Phys Ther.

[26] Feller JA, Bartlett RJ, Lang DM (1996). Patellar resurfacing versus retention in total knee arthroplasty. J Bone Jt Surg Br.

[27] Kujala UM, Jaakkola LH, Koskinen SK, Taimela S, Hurme M, Nelimarkka O (1993). Scoring of patellofemoral disorders. Arthroscopy.

[28] Haim A, Yaniv M, Dekel S, Amir H (2006). Patellofemoral pain syndrome: validity of clinical and radiological features. Clin Orthop Relat Res.

[29] Lee CH, Ha CW, Kim S, Kim M, Song YJ (2013). A novel patellofemoral scoring system for patellofemoral joint status. J Bone Jt Surg Am.

[30] El-Azab H, Halawa A, Anetzberger H, Imhoff AB, Hinterwimmer S (2008). The effect of closed- and open-wedge high tibial osteotomy on tibial slope: a retrospective radiological review of 120 cases. J Bone Jt Surg Br.

